# A brilliant, if imperfect, legacy: In memory of James Watson

**DOI:** 10.1016/j.xinn.2025.101216

**Published:** 2025-12-01

**Authors:** The Innovation editorial team

## Main text

James Watson, one of the most influential scientists of the 20th century, co-discoverer of the double-helix structure of DNA, and laureate of the Nobel Prize in Physiology or Medicine, passed away on November 6, 2025, at the age of 97. His death marks the end of an era in modern biology, yet the scientific revolution he helped ignite continues to define our understanding of life.

**Figure 1:**
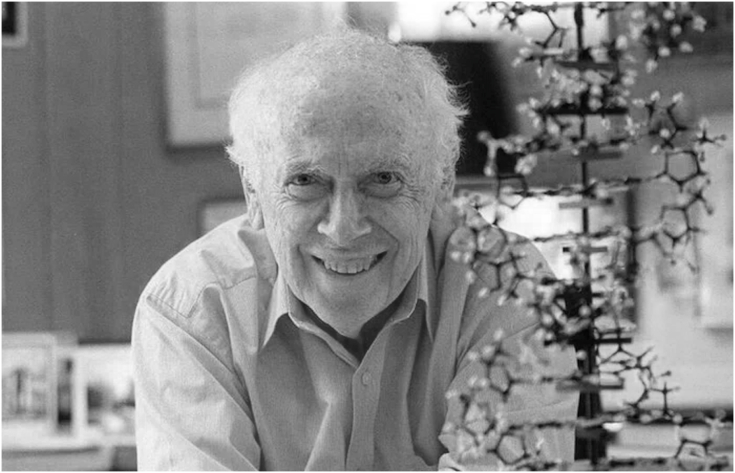
James Watson (1928–2025)

Carrying forward the intellectual lineage of Mendel’s laws of heredity and Darwin’s theory of evolution, the greatest biological breakthrough of the 20th century was undoubtedly the discovery of DNA’s double-helical structure, as published in *Nature* in 1953 by Watson and his colleague Francis Crick. At that moment, humanity was able, for the first time, to glimpse the blueprint of life at the molecular level. The mechanism of heredity encoded in DNA sequences, the nature of genetic variation arising from replication errors, and the profound connection between molecular structure and biological function all became scientifically tractable.

Today, our ability to develop gene therapies, edit genomes, and engineer entirely new organisms—almost like a modern counterpart to the “creator”—traces its roots directly back to the foundational work of Watson and Crick. Their discovery stands as one of the most transformative scientific achievements in human history.

Watson was only 25 years old when he achieved this feat, a time when many scientists have not even begun their doctoral dissertations. Although he and Crick did not receive the Nobel Prize in Physiology or Medicine until 1962, the double-helix discovery had already brought him international acclaim and laid the foundation for the rest of his scientific career.

In 1955, Watson joined Harvard University’s Biology Department as a young faculty member and quickly became a leading force in the emerging field of molecular biology. Under his mentorship, a generation of exceptional young scientists rose to prominence, many of whom would go on to shape the future of life sciences worldwide.

Watson later moved to become director of Cold Spring Harbor Laboratory (CSHL) in New York, bringing with him his emphasis on fostering young researchers and building strong scientific teams. Under his leadership, CSHL evolved into a hub of biological innovation and a gathering place for some of the brightest minds in genetics and molecular biology. Over the decades, the institution produced no fewer than eight Nobel laureates, an extraordinary testament to the scientific ecosystem Watson helped cultivate.

His contributions extended far beyond institutional leadership. Watson was one of the earliest and most vocal advocates for the Human Genome Project. When the project officially began in 1990, he served as its first director, championing its vision and guiding its early progress. Twelve years later, when the sequencing of the human genome was completed, humanity took another monumental leap in understanding its own biological origins and inner workings. This achievement fundamentally reshaped modern medicine and opened the door to precision health, genetic diagnostics, and entirely new therapeutic frontiers.

Watson also played a meaningful role in advancing scientific development in Asia. In 2006, he visited Suzhou, and the following year CSHL established its first overseas branch—Cold Spring Harbor Asia. The institute has since become a premier international platform for scientific exchange, contributing significantly to the growth of life sciences research across the region. For China in particular, Watson’s support left an influence that is still felt today.

And yet, we cannot expect scientists, or any human beings, to be flawless, especially in fields beyond their expertise. In his later years, Watson made several controversial remarks relating to anthropology, intelligence, gender, and race. His suggestion that genetic differences may underlie cognitive differences among groups was sharply criticized by the scientific community. Some of his comments regarding women and Black people were widely condemned for lacking scientific basis and for perpetuating harmful stereotypes. These remarks ultimately led CSHL to revoke his honorary titles.

Such views deserve to be questioned and rejected. But they should not, and cannot, erase Watson’s transformative contributions to biology. The double-helix model, the rise of molecular biology, the early shaping of genetic research institutions, and the initiation of the Human Genome Project are achievements of enduring global significance. They represent a monumental footprint in the history of science—one that cannot be effaced by personal failings or misguided opinions.

Watson was a brilliant scientist, a bold thinker, and at times a provocative figure. He helped redefine the life sciences, trained generations of exceptional researchers, and laid the groundwork for modern genetics, genomics, and biotechnology. Even though he stumbled in areas beyond his scientific domain, his scientific legacy remains towering.

To remember Watson is not to portray him as a perfect figure, but to acknowledge both the brilliance and the flaws, the achievements and the controversies. His life story embodies the complexity of human creativity—a reminder that great scientific breakthroughs often emerge from imperfect individuals.

For all that he gave to science, and for the profound impact his work continues to exert on medicine, biology, and humanity’s understanding of itself, James Watson deserves to be remembered and mourned.

## Declaration of interests

The author declares no competing interests.

